# Supplementary material for: Isoflurane produces antidepressant effects and induces TrkB signaling in rodents
Source: Sci Rep. 2017 Aug 10;7:7811. doi: 10.1038/s41598-017-08166-9 (PMC5552878; doi:10.1038/s41598-017-08166-9)
Supplement: Supplementary file 1 — Supplementary information [file 41598_2017_8166_MOESM1_ESM.pdf]

Supplementary Information for SREP-17-19674

Isoflurane produces antidepressant effects and induces TrkB signaling in rodents

Hanna Antila[a], Maria Ryazantseva[a,b] Dina Popova[a], Pia Sipilä[a], Ramon Guirado[a], Samuel Kohtala[a,b], Ipek Yalcin[c], Jesse Lindholm[a], Liisa Vesa[a], Vinicius Sato[d], Joshua Cordeira[e], Henri Autio[a], Mikhail Kislin[a], Maribel Rios[e], Sâmia Joca[d], Plinio Casarotto[a], Leonard Khiroug[a], Sari Lauri[a,b], Tomi Taira[a,f], Eero Castrén[a]* and Tomi Rantamäki[a,b]*

[a]Neuroscience Center, P.O. Box 56, FI-00014 University of Helsinki, Helsinki, Finland. [b]Division of Physiology and Neuroscience, Department of Biosciences, Faculty of Biological and Environmental Sciences, P.O. Box 66, FI-00014 University of Helsinki, Helsinki, Finland. [c]Institut des Neurosciences Cellulaires et Intégratives, Centre National de la Recherche Scientifique, FR-67084 Strasbourg Cedex, France. [d]School of Pharmaceutical Sciences of Ribeirão Preto 14040-903, Brazil. [e]Tufts University, Boston, MA, USA. [f]Department of Veterinary Biosciences, Faculty of Veterinary Medicine P.O. Box 66, FI-00014 University of Helsinki, Helsinki, Finland.

List of Supplementary Materials

- Detailed description of Materials and Methods
- Complete immunoblots corresponding cropped blot areas shown in main figures
- Supplementary Figures 1–4
- References

Supplementary Materials and Methods

Animals

Adult (~3 months) C57BL/6J, BDNF$^{2L/2LCk\text{-}Cre}$ (Ref [1]), transgenic mice over-expressing flag-tagged TrkB or truncated TrkB.T1[2–4], TrkB$^{flox/-}$:PV-Cre$^{+/-}$ (Refs [5,6]) heterozygous conditional knockout mice with reduced expression of full-length TrkB receptors in parvalbumin positive interneurons and their wild-type littermates were used in the experiments. B6.Cg-Tg(Thy1- YFPH)2Jrs/J mice (Thy1-YFP, Jackson Laboratories, Bar Harbor, ME, USA) expressing yellow fluorescent protein (YFP) in a subpopulation of cortical layer V pyramidal neurons were used for spine density and turnover analysis. Adult male Wistar rats were used for learned helplessness test. The animals were maintained under standard laboratory conditions with free access to food and water. Unless otherwise stated the experiments were performed during the light phase. The experiments were carried out according to the guidelines of the Society for Neuroscience and were approved by the County Administrative Board of Southern Finland (License numbers: ESLH-2007-09085/Ym-23, ESAVI/7551/04.10.07/2013).

Experiments on naïve mice

Pharmacological treatments

Isoflurane (Vetflurane®, Virbac) treatment was induced in a chamber with 4% isoflurane for 2 minutes, after which the mouse freely inhaled isoflurane *via* a mask (3,0 % for 1 min, then 2 % for maximum of 30 min; airflow: 0.3-0.5 l/min)[7]. Sevoflurane anesthesia induction was done with 8% for 2 minutes, and maintained with 4,5-5 % for total of 30 minutes. Halothane anesthesia was done using

similar concentrations and protocol that was used with isoflurane. Body temperature was maintained with a heating pad throughout the treatment. Sham mice were kept in the induction chamber for 2 minutes without isoflurane. NBQX (2,3- Dioxo-6-nitro-1,2,3,4-tetrahydrobenzo[f]quinoxaline-7-sulfonamide, Tocris Bioscience, Bristol, UK) was injected (i.p., 10 mg/kg; dissolved in saline) 10 minutes before sham/isoflurane treatment. Mice were killed by cervical dislocation while still under anesthesia or following the described recovery periods (15 min or 24 h) after stunning with CO_2. Control mice were killed by cervical dislocation after stunning with CO_2.

Immunoprecipitation and western blot

Western blotting analysis was conducted from medial prefrontal cortex (including prelimbic and infralimbic cortices), somatosensory cortex and the whole hippocampus. The brain samples were dissected on a cooled dish and homogenized in NP buffer (137mM NaCl, 20mM Tris, 1% NP-40, 10% glycerol, 48mM NaF, H_2O, Complete inhibitor mix (Roche), 2mM Na_3VO_4). After at least 15-minute incubation on ice, samples were centrifuged (16000*g*, 15 min, +4 °C) and the resulting supernatant collected for further analysis. Sample protein concentrations were measured using Bio-Rad DC protein assay (Bio-Rad Laboratories, Hercules, CA). Flag immunoprecipitation (500 µg protein) was carried out using anti-Flag M2 antibody (F1804; Sigma-Aldrich) as described[8]. Precipitated or unprocessed samples (50 µg protein) were separated with SDS-PAGE under reducing conditions and blotted to a PVDF (polyvinylidene difluoride) membrane (300mA, 1 h, 4°C). Membranes were incubated with the following primary antibodies: anti-p-TrkB^{Y816} (1:1000; kind gift from Dr. Moses Chao, Skirball Institute, NY, USA), anti-p-TrkB^{Y816} (#4168; 1:1000; Cell signaling technology (CST), used in Supplementary Figure 2), anti-p-TrkA/B$^{Y490/Y515}$ (#9141; 1:1000;CST), anti-p-TrkA/B$^{Y674-5/Y706-7}$ (#4621S; 1:1000; CST), anti-TrkB (1:2000; BD Transduction Laboratories, San Jose, CA, USA), anti-p-CREB^{S133} (#9191S; 1:1000; CST), anti-p-Akt^{T308} (#4056S; 1:1000; CST), anti-p-mTOR^{S2481} (#2974S; 1:1000; CST), anti-p-p70S6K$^{T421/S424}$ (#9204S; 1:1000; CST), anti-p-4E-BP1$^{T37/46}$ (#2855; 1:1000; CST), anti-p-GSK3β^{S9} (#9336; 1:1000; CST), anti-p-eEF2^{T56} (#2331; 1:1000; CST), anti-Trk (sc-11; 1:1000; Santa Cruz Biotechnology (SCB)), anti-BDNF (#327-100 (clone 3C11); 1:1000, Icosagen, Estonia), and anti-GAPDH (sc-25778; 1:10000; SCB). Further, the membranes were washed with TBS/0.1% Tween (TBST) and incubated with horseradish peroxidase conjugated secondary antibodies (1:10000 in non-fat dry milk, 1 h at room temperature; Bio-Rad). After subsequent washes, secondary antibodies were visualized using enhanced chemiluminescence (ECL Plus, ThermoScientific, Vantaa, Finland) for detection by Fuji LAS-3000 camera (Tamro Medlabs, Vantaa, Finland).

BDNF ELISA

For the ELISA analyses, NP-lysed brain samples (~100 µg) were acidified and processed as described[9]. BDNF protein levels were quantified using a commercial kit according to instructions (Human BDNF Quantikine ELISA Kit, catalog # DBD00, R&D Systems Europe Ltd., Abingdon, UK) and normalized against total protein content.

qRT-PCR

Total *Bdnf* mRNA levels were measured from hippocampus samples using quantitative RT-PCR as described[10]. Briefly, extracted total RNA was treated with DNAse I mix (Fermentas GmbH, Helsinki,

Finland) and then reverse transcribed using oligo(dT) primer and RevertAid First Strand cDNA synthesis kit (Thermo Scientific). The control reactions without reverse transcriptase were also performed. The amount of cDNA was quantified using Maxima SYBR green qPCR master mix (Thermo Scientific) by real-time PCR. Total *Bdnf* cDNA was amplified using the following primers: 5´- GAAGGCTGCAGGGGCATAGACAAA-3´ and 5´-TACACAGGAAGTGTCTATCCTTATG-3´. For normalization GAPDH cDNA levels were analyzed with the following primers 5´-GGTGAAGGTCGGTGTGAACGG-3´ and 5´-CATGTAGTTGAGGTCAATGAAGGG-3´. C_t values from each sample were obtained using the LightCycler 480 software (Roche Diagnostics Ltd.).

Spine analysis from fixed tissue

The density of dendritic spines was analyzed in fixed cortical sections from B6.Cg-Tg(Thy1-YFPH)2Jrs/J (Thy1-YFP, Jackson Laboratories, Bar Harbor, ME, USA) mice. Animals were transcardially perfused at 24 hours after isoflurane/sham treatment under deep pentobarbital anesthesia with 4% PFA in PB 0.1M and post-fixed for 1 hour. Floating sections (50 µm thick) were cut using a vibratome (VT 1000S, Leica, Germany) and processed for immunohistochemistry as follows: PBS wash, blocking for 1 h (10% normal goat serum in PBS/0,2% Triton-X100), anti-YFP antibody overnight (chicken polyclonal 1:1000; Abcam), PBS wash, Alexa-488-conjugated goat secondary antibody for 2 h (1:200; Invitrogen), PB wash and mounting using fluorescence medium (Dako). Images were obtained using a confocal microscope (Leica TCS SP5II HCS), and pyramidal neurons from the medial prefrontal cortex (mPFC) and somatosensory cortex (SSCx) were selected with the following criteria: intense fluorescence, soma located in layer V, and primary apical dendrite >200 µm long. We imaged the dendrites in three ~65 µm long segments (proximal/medial/distal). We distinguished different types of dendritic spines: (i) stubby (protrusion length <1.5 µm); (ii) mushroom (clearly visible head with a diameter >1.5 times the average length of the neck, and the total length of the protrusion <3 µm) and; (iii) filopodia/thin (the length of the protrusion >3 µm or non-headed 1.5-3 µm protrusion). Similarly, we analyzed extra-distal dendritic segments in the hippocampus and mPFC (see # symbol in **Figure 5**). The analysis was done by a person blind to the treatment.

In vivo two-photon microscopic imaging in awake mice

B6.Cg-Tg(Thy1- YFPH)2Jrs/J (Thy1-YFP, Jackson Laboratories, Bar Harbor, ME, USA) mice were used for the analysis of dendritic spine turnover in an awake *in vivo* imaging experiment. A cranial window with a 4 mm diameter circular glass coverslip was implanted onto the skull above the somatosensory cortex (SSCx) under a combination anesthesia of fentanyl (0.05 mg/kg), midazolam (5 mg/kg) and medetomidine (0.5 mg/kg) (i.p.). Three weeks post-surgery and one week prior to imaging the mice were trained for eight 2-hour sessions with their head fixed but otherwise freely moving in an airlifted flat-floored Mobile HomeCage (Neurotar Ltd., Finland). For imaging, animals were intravenously injected with Texas Red –tagged 70 kDa dextran tracer and placed under the Fluo View 1000MP multiphoton microscope (Olympus, Hamburg, Germany). Blood vessels were used to locate the selected dendritic segments throughout the imaging sessions. Stacks of the YFP-expressing dendrites displaying spines from layer 1 in the SSCx were acquired. Five different dendritic segments from 4 different areas were analyzed per animal to study the time-lapse spine turnover (n indicating the number of dendritic segments; see Ref. [11]). The spine formation and elimination rates were calculated

as the number of spines that have appeared or disappeared, respectively at a given time point compared to the previous time point relative to the total number of spines present at that or the previous time, respectively.

Immunohistochemistry and analysis of FOSB intensity in parvalbumin and somatostatin neurons

The tissue was processed as free-floating sections, washed in PBS, then incubated in 10% normal donkey serum (NDS; Gibco), 0.2% Triton-X100 (Sigma) in PBS for 1h. Sections were then incubated for 48h at 4°C with a cocktail containing IgG mouse anti-Somatostatin (1:1000; Swant), IgG guinea pig anti-Parvalbumin (1:2000; Swant) and IgG rabbit anti-FosB (1:500; Santa Cruz) diluted in PBS 0.2% Triton-X100. After washing again with PBS, sections were incubated for 2h at room temperature with a cocktail containing the following secondary antibodies: anti-mouse IgG conjugated with Alexa 488, anti-guinea pig IgG conjugated with A546 and anti-rabbit IgG conjugated with Alexa 647 (1:200; Life Technologies) diluted in 0.2% Triton-X100 in PBS. Sections were then washed in PB 0.1M, mounted on slides and coverslipped using fluorescence mounting medium (Dako).

Image stacks from at least three sections per brain area per mouse were obtained using a Zeiss LSM 710 confocal microscope with a 20x objective. Single confocal planes (pinhole = 1AU) from the same depth of the tissue (2-3 μm below the upper surface) were processed using FIJI to analyze the expression fluorescence intensity of FosB in neurons. A macro function was written to automatically threshold the 1% of pixels with highest fluorescence intensity in parvalbumin or somatostatin channel. We checked that these values effectively overlapped the profile of neurons expressing parvalbumin or somatostatin. Then, we used these profiles to mask the original image and analyzed the fluorescence intensity of FosB specifically within these subpopulations of interneurons. Finally, we subtracted the interneuron profiles from our original images and analyzed the remaining neurons with the 5% highest FosB fluorescence intensity, which therefore must consist predominantly of excitatory pyramidal neurons.

Electrophysiological recordings

At 24 hour after a 30 min isoflurane treatment, hippocampal slices (400 μm) were cut with a vibratome as described[12]. The slices were used 1-4 hours after cutting. After at least 1 hour recovery period at room temperature, the slices were transferred into an interface-type recording chamber (32°C) perfused with artificial cerebrospinal fluid (aCSF; in mM: 124 NaCl, 3 KCl, 1.25 NaH_2PO_4, 1 $MgSO_4$, 26 $NaHCO_3$, 15 D-glucose, 2 $CaCl_2$) bubbled with the mixture of 5% CO_2/95% O_2 and applied with the rate of 1 ml/min. Extracellular recordings from CA1 stratum radiatum were obtained with aCSF-filled glass microelectrodes (2–5 MΩ) using an Axopatch 200B amplifier (Axon Instruments, Union City, CA, USA). Field excitatory potentials (fEPSP) were evoked with a bipolar stimulating electrode placed in the Schaffer collateral pathway. Baselines were recorded for 20-30 minutes (stimulation frequency was 0.05 Hz, pulse duration 0.1 ms) and post-induction responses were normalized to the final 10 min of the baseline. After input–output data was collected the stimulus intensity was adjusted to evoke half-maximal fEPSP amplitude. LTP was induced by 100-Hz tetanic stimulation for 1 s. The level of LTP was measured as a percentage increase of the fEPSP slope, averaged at a 1-min interval 30 min after the tetanus, and compared to the averaged baseline fEPSP slope. For paired-pulse stimulation, interpulse intervals from 10 to 200 ms were tested. To antagonize fast $GABA_A$ synaptic transmission

picrotoxin (PiX; 100 µM) was used. WinLTP (0.95b or 0.96, www.winltp.com) was used for data acquisition[13]. To investigate inhibitory postsynaptic currents, 25 µM MK-801, 5 µM L-689-560 and 20 µM NBQX were included in the aCSF to antagonize NMDA and AMPA receptors. Whole cell voltage clamp recordings (-70 mV) from CA1 neurons were obtained with glass microelectrodes (4.5 - 6 MOhm) filled with a solution containing the following (in mM): 130 CsCl, 10 HEPES, 0.5 EGTA, 4 Mg-ATP, 0.3 Na-GTP, 5 QX 314 and 8 NaCl (285 mOsm, pH 7.2) using a Multiclamp 700A amplifier (Axon Instruments, Union City, CA, USA). To record miniature (action potential independent) IPSCs, 1 µM TTX was added to aCSF. The data analysis was made with Mini Analysis Program, version 5.6.6 (Synaptosoft, Decatur, GA). The person conducting the electrophysiological experiments and analysis was blind to the treatment groups.

Forced swim test

For the forced swim test a mouse was placed into a glass cylinder (diameter 19 cm, height 24 cm) filled with water (21±1°C) to the height of 14 cm. The latency to the first immobility period (passive floating, when the animal was motionless or doing only slight movements with tail or one hind limb) and overall immobility was measured during the 6-minute testing period. The mice were tested in FST 15 minutes after the isoflurane anesthesia. The analysis was done from video recordings by a person blind to the treatment and to the genotype of the animals.

Open field test

The open field test was used to assess general locomotor activity. The test was performed for 30 min in an illuminated (300 lux) transparent acrylic cage (length 28.5 × height 8.5 × width 20 cm) (Med Associates). Interruptions of infrared photo beams were used to calculate the overall distance traveled (cm).

Learned helplessness model of depression

Animals

Male Wistar rats weighing 240-260 g at the beginning of each experiment were housed alone in a temperature controlled room (24±1°C) under standard laboratory conditions with free access to food and water and a 12 h light/12 h dark cycle (lights on at 7:00 a.m.). Procedures were conducted in conformity with the Brazilian Society of Neuroscience and Behavior guidelines for the care and use of laboratory animals, which are in compliance with international laws and politics.

Isoflurane treatment

A cylindrical chamber was used to the administration of isoflurane (30 cm high, 18,5 cm of diameter). A suspended wad of cotton with 3 ml of isoflurane was used to induce anesthesia primarily. After 1 minute inside the chamber the rats were maintained under observation and anesthetized for total duration of 30 minutes (counting from the moment they entered in the chamber) using a cylindrical tube mask with a little wad of cotton. 0.1 ml of isoflurane was administered to the wad every five minutes, till the end of the 30 minutes.

Learned Helplessness

The Learned Helplessness test was performed as described[14]. The protocol includes a pre-test session (PT) with 40 unescapable footshocks (0.4 mA, per 10 s) and on the 7th day a test session (T) in which 30 escapable footshocks (0.4 mA, per 10 s) are preceded (5 s) by an alarm tone (60 dB, 670 Hz). In the

test session the shock could be interrupted or avoided by the animal if it crosses to the other compartment of the chamber during the tone presentation or during the footshock application. On day 2 (24 h after the PT) the animals were anesthetized with isoflurane for 30 min in a chamber and the control animals were exposed (1 minute) to the same chamber without any trace of isoflurane.

Neuropathic pain model of depression

Animals

Behavioral experiments were conducted in male C57BL/6J mice (Charles River, L'Arbresle, France). Experiments started with 8 to 9 weeks old mice, group-housed five per cage and kept under a 12-hour light/dark cycle (lights on at 10:00 PM). Food and water were available *ad libitum*. Animal facilities are registered for animal experimentation (Agreement C67-482-1). All procedures were performed in accordance with guidelines for animal experimentation of the International Association for the Study of Pain (IASP) and European Communities Council Directive 86/6609/EEC and approved by the local ethical committee of the University of Strasbourg (CREMEAS, n°AL-04).

Isoflurane administration

Isoflurane (Aerrane®, Baxter, France) in oxygen (2 l/min) was administered to the mice in their home cages to diminish the potential stress effect induced by a new environment. In the first 2 minutes, the mice were subjected to isoflurane 4%. The concentration of drug was decreased progressively to 3% for 2 min, then to 2% for 8 min and finally to 1% for 18 min. The control group was monitored in the same room for 30 min without receiving isoflurane. All cages were modified with a ceiling adapted for isoflurane administration.

Induction of neuropathic pain

Chronic neuropathic pain was induced by placing a cuff around the main branch of the right sciatic nerve[15,16]. Before surgery, mice were assigned to the experimental groups so that these groups did not initially differ for the mechanical nociceptive threshold or for the body-weight. Surgery was performed under ketamine (17 mg/ml)/xylazine (2.5 mg/ml) anesthesia (intraperitoneal, 4 ml/kg) (Centravet, Taden, France). The common branch of the right sciatic nerve was exposed and a 2 mm section of split PE-20 polyethylene tubing (Harvard Apparatus, Les Ulis, France) was placed around it for Cuff group (Neuropathic group). Sham-operated mice underwent the same procedure without cuff implantation (Sham group).

Nociceptive test

The mechanical threshold of hindpaw withdrawal was evaluated using von Frey filaments and the results were expressed in grams (Bioseb, Chaville, France)[16,17]. Mice were placed in clear Plexiglas® boxes (7 x 9 x 7 cm) on an elevated mesh screen. They were allowed to habituate for 15 min before testing. Calibrated von Frey filaments were applied to the plantar surface of each hindpaw until they just bend, in a series of ascending forces up to the mechanical threshold (0.4 to 10 grams). Each filament was tested five times per paw and the threshold was defined as 3 or more withdrawals observed out of the 5 trials[15,18].

Novelty suppressed feeding (NSF) test: We performed the NSF test at 12th hour after the isoflurane administration during the dark phase, under red light. The testing apparatus consisted of a 40 x 40 x 30 cm plastic box with the floor covered with 2 cm of sawdust. Twenty-four hours prior to test, food was

removed from the home cage. At the time of testing, a single pellet of food was placed on a paper in the center of the box. Then, an animal was placed in a corner of the box and latency to eat the pellet was measured within a 5 minute period[15]. This test induces a conflict between the drive to eat the pellet and the fear of venturing in the center of the box[19].

Statistical analysis

Results are represented as mean ± SEM (standard error of mean) unless otherwise stated. For statistical analysis two-sided tests including unpaired two-tailed Student's t-test, Mann Whitney U test (non-normally distributed data), one-way analysis of variance (ANOVA), mixed model ANOVA, two-way ANOVA, Pearson's χ^2 test and Spearman's correlation test were used. Levene's test was used to define the equality of variances. If the variances differed significantly non-parametric test was used. *Post hoc* analysis was conducted with Tukey HSD or Dunnett's test. Statistically significant p value was set to $\leq$ 0.05. Outliers were considered as values differing more than 2x standard deviation from the mean of the group. To calculate the proper sample sizes we used power calculations (α=0.05 and power 0.80) and estimated the standard deviations and effect sizes based on our previous experience and literature.

Supplementary complete immunoblots corresponding cropped blot areas shown in main figures

Complete western blots where the cropped blot areas shown in main Figures 2, 3 and 4 have been marked with red quadrangles. The protein of interest is indicated with an arrow. Some of the western blots have been combined (overlaid) with a picture of the protein marker to demonstrate the molecular weights of the quantitated proteins (Figure 2A, 2B, 2D, 3A, 3B, 3C, 4A, 4B, 4C). The cropped area in the Figure 2B has been reversed in the main figure (marked with two-headed arrow). Abbreviations: kDa, kilodalton; M, protein size marker; C, control treatment; I, isoflurane treatment; -, empty lane; S, subanesthetic isoflurane treatment (0,25%); X, unrelated sample; 2, 2 minutes isoflurane treatment; N, NBQX treatment; NI, NBQX and isoflurane treatments; Trk, tropomyosin-related kinase; AKT, protein kinase B; CREB, cAMP response element binding protein; mTor, mammalian target of rapamycin; P70S6K, ribosomal protein S6 kinase beta-1; 4EBP1, eukaryotic translation initiation factor 4E-binding protein 1; GSK3, glycogen synthase kinase 3; GAPDH; glyceraldehyde 3-phosphate dehydrogenase; eEF2, eukaryotic elongation factor 2; BDNF, brain-derived neurotrophic factor; KO, BDNF conditional knock out.

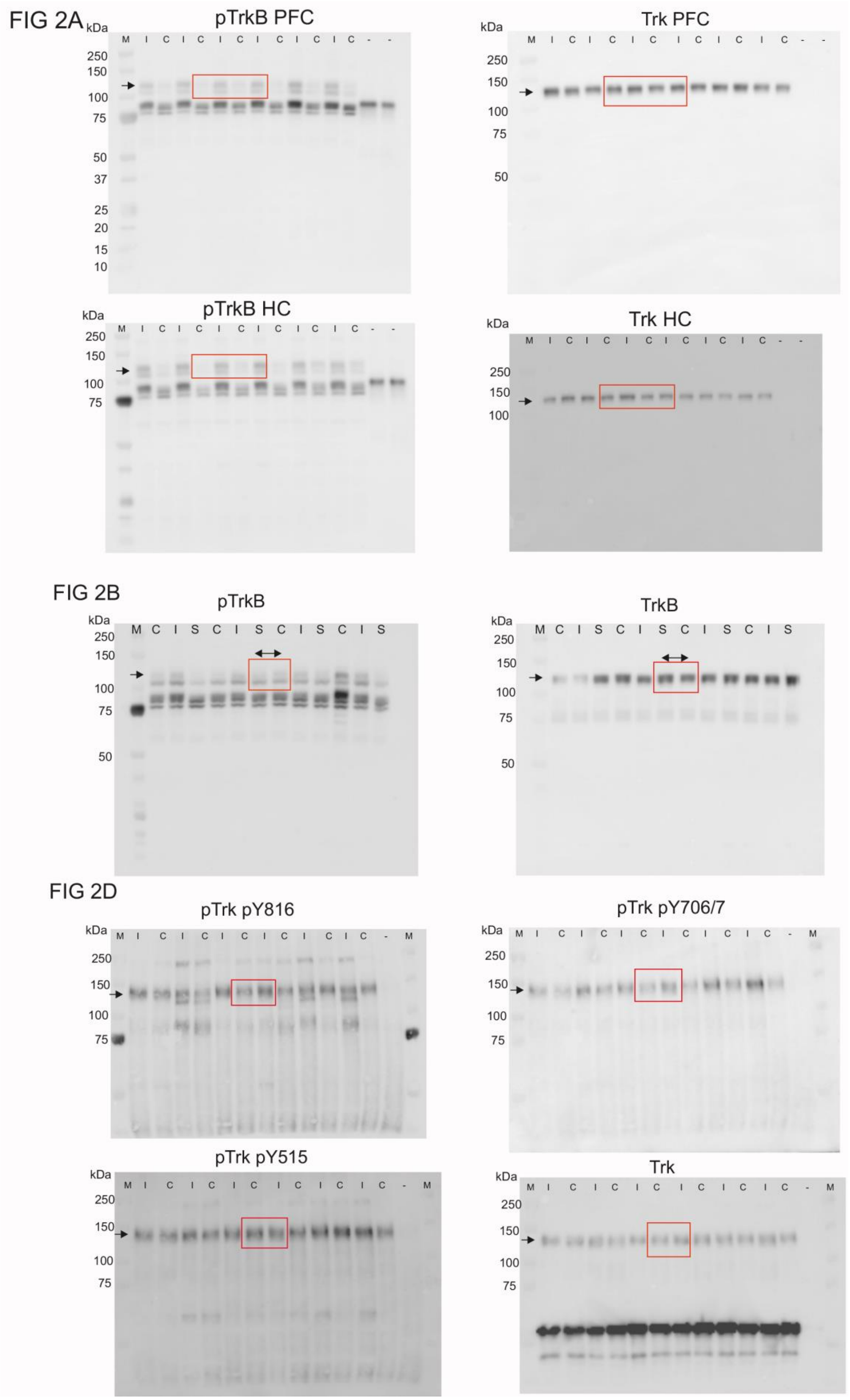
FIG 2A
pTrkB PFC
Trk PFC
pTrkB HC
Trk HC
FIG 2B
pTrkB
TrkB
FIG 2D
pTrk pY816
pTrk pY706/7
pTrk pY515
Trk
kDa
250
150
100
75
50
37
25
20
15
10

Fig 3A

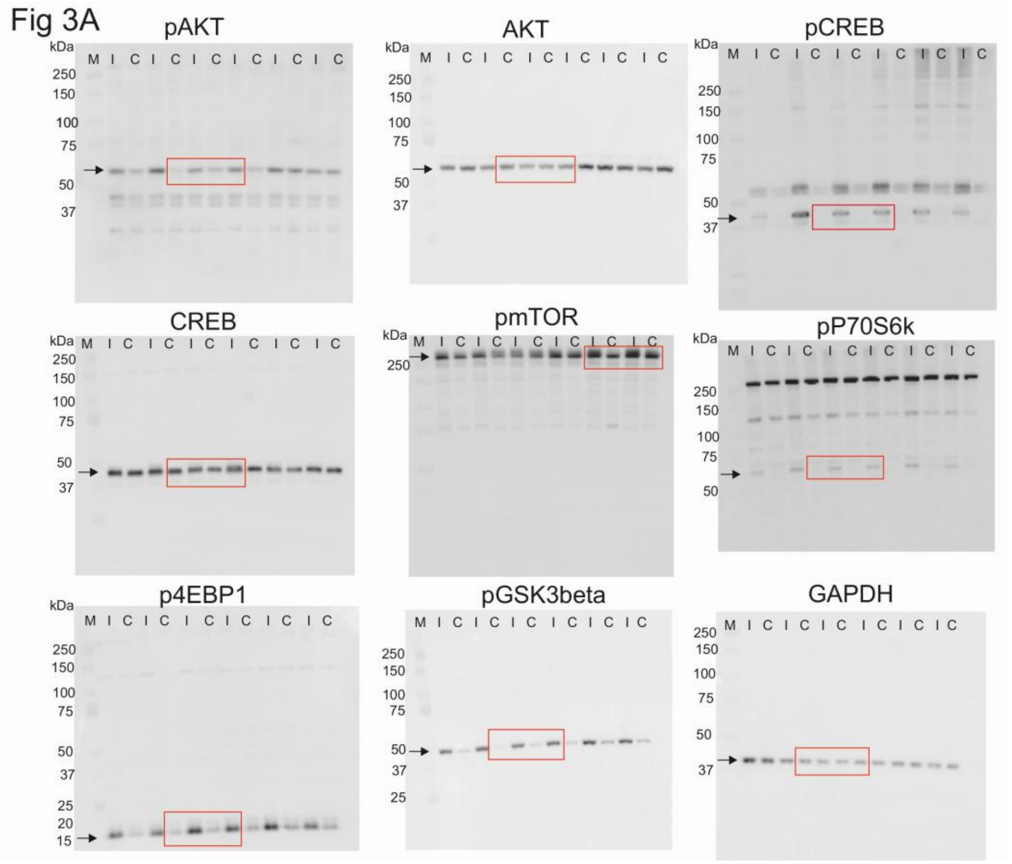
pAKT
AKT
pCREB
CREB
pmTOR
pP70S6k
p4EBP1
pGSK3beta
GAPDH
kDa

Fig 3B

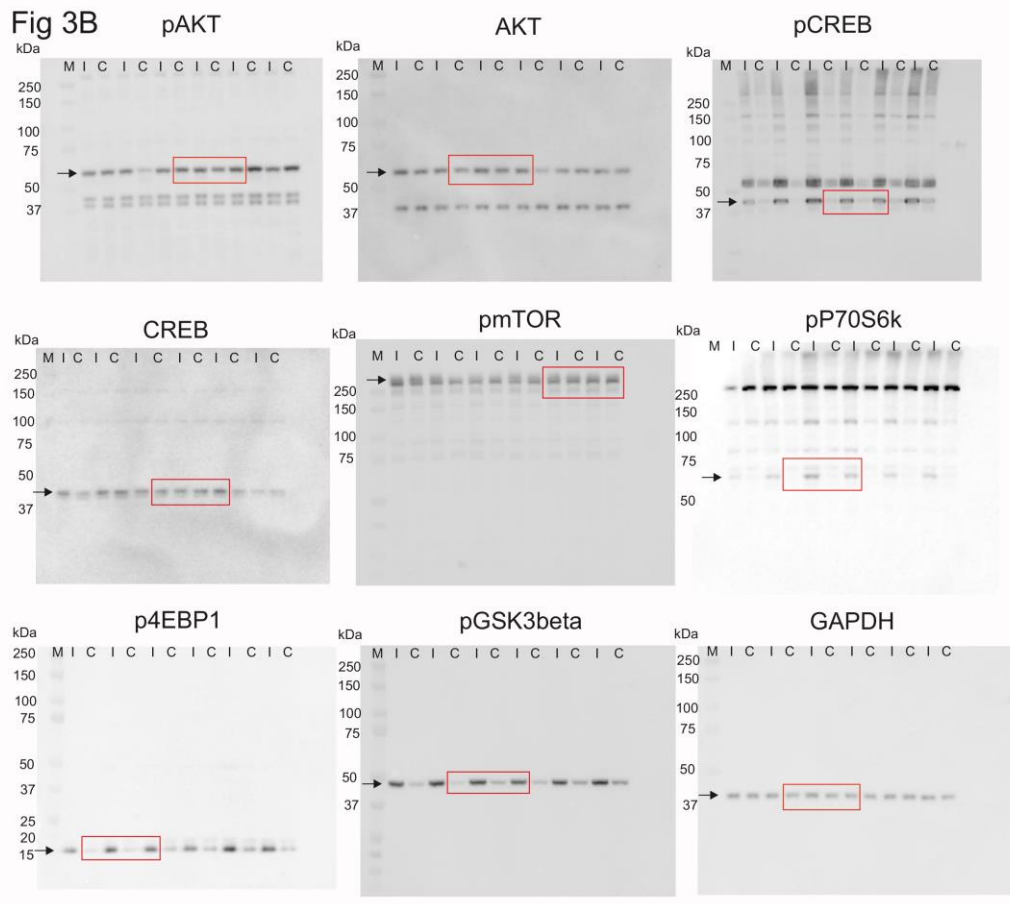
pAKT
AKT
pCREB
CREB
pmTOR
pP70S6k
p4EBP1
pGSK3beta
GAPDH
kDa

Fig 3C

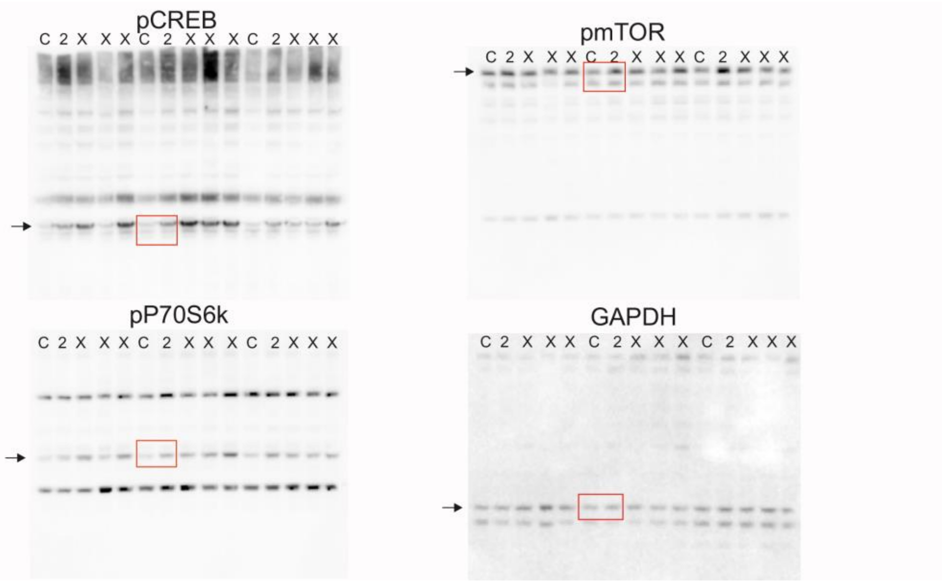

FIG 4A

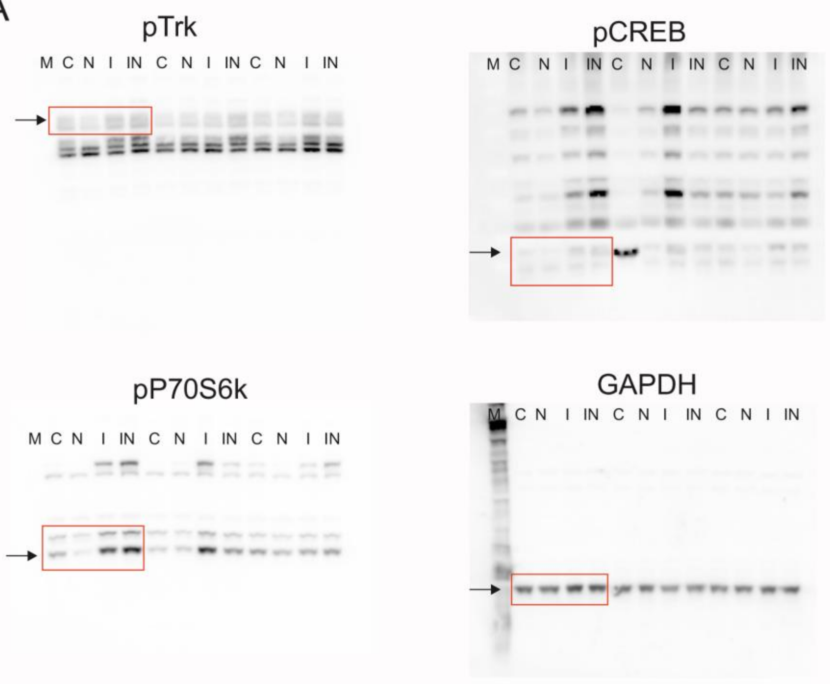

FIG 4B

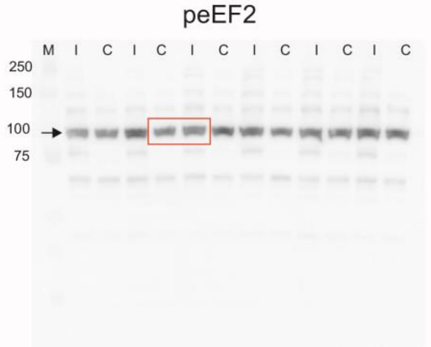

FIG 4C

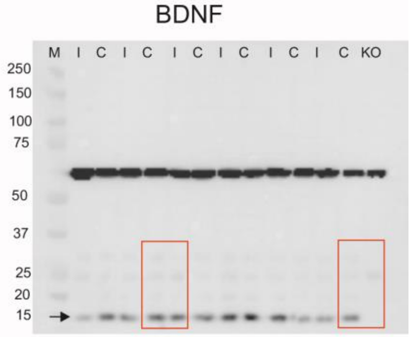

Supplementary Figures

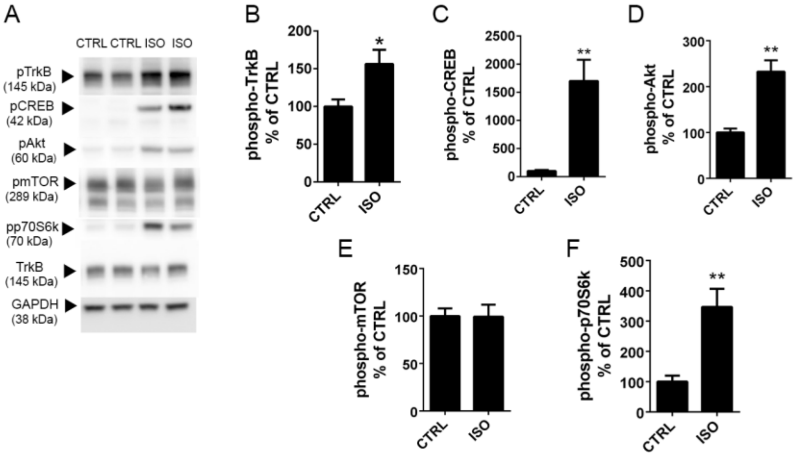

Supplementary Figure 1: Isoflurane anesthesia activates TrkB signaling in the adult mouse somatosensory cortex. (**A**) Representative western blots showing the effects of 30 min isoflurane administration on phosphorylations of (**B**) $TrkB^{Y816}$ (p=0.0029), (**C**) $CREB^{S133}$ (p=0.0017), (**D**) Akt^{T308} (p=0.0022), (**E**) $mTOR^{S2481}$ (p=0.9639) and (**F**) $p70S6K^{T421/S424}$ (p=0.0043). TrkB phosphorylation levels have been normalized to total TrkB whereas other phospho-proteins to GAPDH. n=6/group. *p<0.05, **p<0.01; Student's t test (B,C,E), Mann Whitney U test (D,F). Abbreviations: CTRL, control treatment; ISO, isoflurane anesthesia; CREB, cAMP response element binding protein; Akt, protein kinase B; mTOR, mammalian target of rapamycin**.**

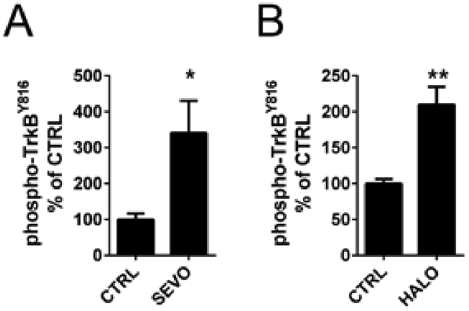

Supplementary Figure 2: Sevoflurane and halothane anesthesias activate TrkB. Phosphorylation of $TrkB^{Y816}$ was significantly increased after 30 minutes treatment with (**A**) sevoflurane (p=0.0254; n=6/group) and (**B**) halothane (p=0.0057; n=4/group). pTrkB levels normalized to total TrkB levels. *p<0.05, **p<0.01; Student's t test. Abbreviations: CTRL, control treatment; SEVO, sevoflurane treatment; HALO, halothane treatment.

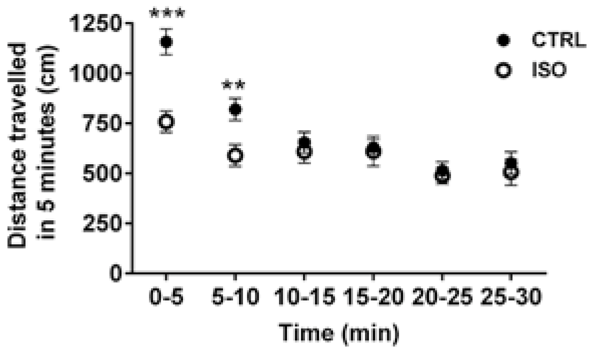

Supplementary Fig. 3: Isoflurane reduces locomotor activity. Isoflurane treatment (30 min) significantly reduces overall locomotor activity in the open field test during the first 10 minutes (between subjects mixed model ANOVA for treatment effect ($F_{1,28}$=5.403, p=0.028); t-test at 0-5min CTRL vs ISO p<0.001, at 5-10min CTRL vs ISO p=0.006). The open field test was started after a recovery period of 15 minutes to assess the locomotor activity at the same time point at which the forced swim test was done. (n=15/group) **p<0.01, ***p<0.001; Mixed model ANOVA followed by Bonferroni corrected Student t-test. Abbreviations: CTRL, control treatment; ISO, isoflurane treatment.

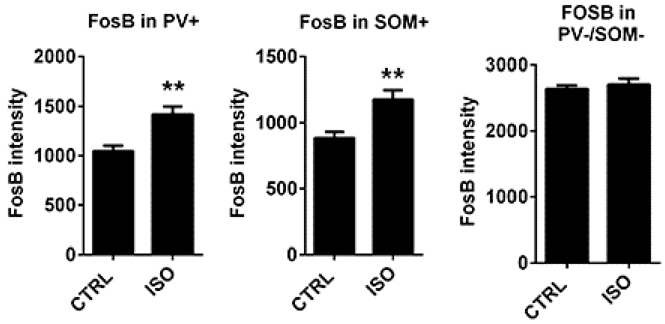

Supplementary Fig 4: FosB intensity in the medial prefrontal cortex. Isoflurane anesthesia (30 min) 24 h before the tissue collection increased FosB intensity specifically in the parvalbumin (PV+, p=0.0011) and somatostatin positive (SOM+, p=0.0025) cells of the PFC. The FosB intensity was not increased in cells not expressing parvalbumin and somatostatin (PV-/SOM-). **p<0.01, Student's t test.

References

1. Rios, M. *et al.* Conditional deletion of brain-derived neurotrophic factor in the postnatal brain leads to obesity and hyperactivity. *Mol. Endocrinol. Baltim. Md* **15,** 1748–1757 (2001).
2. Koponen, E. *et al.* Transgenic mice overexpressing the full-length neurotrophin receptor trkB exhibit increased activation of the trkB-PLCgamma pathway, reduced anxiety, and facilitated learning. *Mol. Cell. Neurosci.* **26,** 166–181 (2004).
3. Saarelainen, T. *et al.* Transgenic mice overexpressing truncated trkB neurotrophin receptors in neurons have impaired long-term spatial memory but normal hippocampal LTP. *Synap. N. Y. N* **38,** 102–104 (2000).
4. Saarelainen, T. *et al.* Transgenic mice overexpressing truncated trkB neurotrophin receptors in neurons show increased susceptibility to cortical injury after focal cerebral ischemia. *Mol. Cell. Neurosci.* **16,** 87–96 (2000).
5. Hippenmeyer, S. *et al.* A developmental switch in the response of DRG neurons to ETS transcription factor signaling. *PLoS Biol.* **3,** e159 (2005).
6. Minichiello, L. *et al.* Essential role for TrkB receptors in hippocampus-mediated learning. *Neuron* **24,** 401–414 (1999).
7. Kohtala, S. *et al.* Brief Isoflurane Anesthesia Produces Prominent Phosphoproteomic Changes in the Adult Mouse Hippocampus. *ACS Chem. Neurosci.* **7,** 749–756 (2016).
8. Rantamäki, T. *et al.* Pharmacologically diverse antidepressants rapidly activate brain-derived neurotrophic factor receptor TrkB and induce phospholipase-Cgamma signaling pathways in mouse brain. *Neuropsychopharmacol. Off. Publ. Am. Coll. Neuropsychopharmacol.* **32,** 2152–2162 (2007).
9. Karpova, N. N. *et al.* Darkness reduces BDNF expression in the visual cortex and induces repressive chromatin remodeling at the BDNF gene in both hippocampus and visual cortex. *Cell. Mol. Neurobiol.* **30,** 1117–1123 (2010).
10. Rantamäki, T. *et al.* The Impact of Bdnf Gene Deficiency to the Memory Impairment and Brain Pathology of APPswe/PS1dE9 Mouse Model of Alzheimer's Disease. *PLoS ONE* **8,** e68722 (2013).
11. Chen, J. L. *et al.* Clustered dynamics of inhibitory synapses and dendritic spines in the adult neocortex. *Neuron* **74,** 361–373 (2012).

12. Pavlov, I., Rauvala, H. & Taira, T. Enhanced hippocampal GABAergic inhibition in mice overexpressing heparin-binding growth-associated molecule. *Neuroscience* **139,** 505–511 (2006).
13. Anderson, W. W. & Collingridge, G. L. Capabilities of the WinLTP data acquisition program extending beyond basic LTP experimental functions. *J. Neurosci. Methods* **162,** 346–356 (2007).
14. Joca, S. R. L., Padovan, C. M. & Guimarães, F. S. Activation of post-synaptic 5-HT(1A) receptors in the dorsal hippocampus prevents learned helplessness development. *Brain Res.* **978,** 177–184 (2003).
15. Yalcin, I. *et al.* A time-dependent history of mood disorders in a murine model of neuropathic pain. *Biol. Psychiatry* **70,** 946–953 (2011).
16. Bohren, Y. *et al.* Antidepressants suppress neuropathic pain by a peripheral β2-adrenoceptor mediated anti-TNFα mechanism. *Neurobiol. Dis.* **60,** 39–50 (2013).
17. Yalcin, I. *et al.* beta(2)-adrenoceptors are critical for antidepressant treatment of neuropathic pain. *Ann. Neurol.* **65,** 218–225 (2009).
18. Barrot, M. Tests and models of nociception and pain in rodents. *Neuroscience* **211,** 39–50 (2012).
19. Santarelli, L. *et al.* Requirement of hippocampal neurogenesis for the behavioral effects of antidepressants. *Science* **301,** 805–809 (2003).
